# Addition of an N-Terminal Poly-Glutamate Fusion Tag Improves Solubility and Production of Recombinant TAT-Cre Recombinase in *Escherichia coli*

**DOI:** 10.4014/jmb.1909.09028

**Published:** 2019-11-04

**Authors:** A-Hyeon Kim, Soohyun Lee, Suwon Jeon, Goon-Tae Kim, Eun Jig Lee, Daham Kim, Younggyu Kim, Tae-Sik Park

**Affiliations:** 1 Department of Life Sciences, Gachon University, Sungnam 320, Republic of Korea; 2 Department of Research and Development, LumiMac, Inc., Seoul 05844, Republic of Korea; 3 Department of Internal Medicine, Yonsei University College of Medicine, Seoul 0722, Republic of Korea

**Keywords:** Cre recombinase, inclusion body, solubility, polyglutamate, trans-activator of transcription

## Abstract

Cre recombinase is widely used to manipulate DNA sequences for both in vitro and in vivo research. Attachment of a trans-activator of transcription (TAT) sequence to Cre allows TATCre to penetrate the cell membrane, and the addition of a nuclear localization signal (NLS) helps the enzyme to translocate into the nucleus. Since the yield of recombinant TAT-Cre is limited by formation of inclusion bodies, we hypothesized that the positively charged arginine-rich TAT sequence causes the inclusion body formation, whereas its neutralization by the addition of a negatively charged sequence improves solubility of the protein. To prove this, we neutralized the positively charged TAT sequence by proximally attaching a negatively charged poly-glutamate (E12) sequence. We found that the E12 tag improved the solubility and yield of E12-TAT-NLS-Cre (E12-TAT-Cre) compared with those of TAT-NLS-Cre (TATCre) when expressed in *E. coli*. Furthermore, the growth of cells expressing E12-TAT-Cre was increased compared with that of the cells expressing TAT-Cre. Efficacy of the purified TATCre was confirmed by a recombination test on a floxed plasmid in a cell-free system and 293 FT cells. Taken together, our results suggest that attachment of the E12 sequence to TAT-Cre improves its solubility during expression in *E. coli* (possibly by neutralizing the ionic-charge effects of the TAT sequence) and consequently increases the yield. This method can be applied to the production of transducible proteins for research and therapeutic purposes.

## Introduction

A cell-penetrating peptide (CPP), also called a protein transduction domain (PTD) or Trojan peptide, allows for efficient intracellular delivery of various proteins with rare toxicity [1]. CPPs are oligopeptides 5 to 30 amino acid residues long, for example, TAT_47–57_ (YGRKKRRQRRR) from HIV-1, polyarginine 9 (RRRRRRRRR), and penetratin43–57 (RQIKIWFQNRRMKWKK) [2-4]. TAT, trans-activator of transcription, is a basic peptide (arginine and lysine) and serves as a PTD [5]. The amphipathic and heavily cationic TAT_47–57_ is internalized into the cell through receptor-independent, lipid raft–dependent micropinocytosis [6]. The cargo, transferred to the cytoplasm by the TAT PTD, is then translocated into the nucleus because of a nuclear localization sequence (NLS) from SV-40 large T antigen [7, 8].

Various proteins containing the cationic CPP tag sequence have been produced in *E. coli* [8]. Nevertheless, in some cases, the expression of these proteins in *E. coli* creates insoluble protein aggregates known as inclusion bodies in the cytoplasm or periplasm when the expressed foreign protein constitutes more than 2% of total cellular protein [9]. To produce functionally active proteins, the inclusion bodies need to be purified, and a cumbersome process is required to recover a native-like conformation: isolation of inclusion bodies, solubilization by denaturation with chaotropic agents, refolding, and purification of the soluble protein from the refolding reaction [10]. Some amount of the insoluble protein is correctly refolded, and the degree of correct folding is dependent upon the nature of the protein sequence as well as the choice of a refolding procedure [10, 11]. A surfactant or a high concentration (2 to 8 M) of urea or guanidine hydrochloride as a denaturing agent is generally added to release the aggregated protein by recovering its conformation [12-14].

Cre recombinase has been widely employed to induce site-specific gene recombination or regulate the expression of a gene of interest in vitro or in vivo [15, 16]. Cre recognition sites, two loxP sites, each consisting of 34 bp, flank the targeted site called a “floxed allele”. Cre-mediated recombination causes fusion of two flanked loxP sites and releases this DNA part [17]. Furthermore, a floxed reporter gene such as β-galactosidase or a fluorescent protein in the cell visually shows the effect of Cre [16, 18]. TAT-fused Cre has been used to improve penetration of Cre into cells [15, 16, 18].

Previously, the amount of expressed TAT-Cre has been less than that of nonfused Cre, but the solubility of nonfused Cre has been relatively higher than that of TAT-Cre [19]. Judging by these findings, we reasoned that the heavily cationic TAT could be a cause of the low solubility of TAT-Cre in *E. coli* cytosol, whereas neutralization of the charge by proximal introduction of a negatively charged amino acid sequence could enhance the solubility.

In this study, we tested whether addition of a negatively charged poly-glutamate (E12) sequence can improve solubility of TAT-Cre and the production yield in *E. coli*. Once the E12-tagged TAT-Cre was purified and treated with TEV protease, highly pure and active TAT-Cre was obtained, and its yield was greater in comparison with the expression of TAT-Cre alone.

## Materials and Methods

### Construction of Prokaryotic Plasmids

pETM11 contains the T7 promoter followed by a His tag (H6) and a TEV protease recognition site (TEV site). The E12 (EEEEEGSEEEEEEE)-encoding oligonucleotides were inserted between H6 and the TEV site of pETM11 by PCR-based in vitro mutagenesis. The TAT peptide (GRKKRRQRRRPPAGTSVSL)-encoding DNA fragment, NLS (KKKRKV)-encoding fragment, and the Cre-encoding fragment were assembled at the C terminus of the TEV site by PCR. We confirmed the correct structure of the plasmid by sequencing.

### Expression and Purification of the Recombinant Protein

Cre expression plasmids, H6-TAT-Cre or H6-E12-TAT-Cre, were transfected into BL21(DE3) cells, and the latter were grown overnight in 1 L of the Luria–Bertani (LB) medium supplemented with 40 μg/ml kanamycin. Expression of recombinant proteins was induced with 0.5 mM isopropyl β-D-1-thiogalactopyranoside (IPTG) at 37°C for 3 h. The cell growth was measured every hour as optical density at 600 nm (OD_600_) for 10 h. The cells were harvested by centrifugation and were sonicated with lysis buffer (5 mM imidazole, 500 mM NaCl, 5% of glycerol, 4 mM β-mercaptoethanol, and 20 mM Tris-HCl pH 8.0). The lysates of cells were centrifuged at 15,000 g for 20 min at 4°C to obtain both the supernatant and pellet (containing inclusion bodies). Each His-tagged protein in the supernatant was purified via Ni-NTA Agarose Beads (Qiagen), which were washed with wash buffer (20 mM imidazole, 500 mM NaCl, 5% glycerol, 4 mM β-mercaptoethanol, 0.05% of Tween 20, and 20 mM Tris-HCl pH 8.0). His-tagged TAT-Cre and E12-TAT-Cre were eluted with elution buffer (250 mM imidazole, 500 mM NaCl, 5% of glycerol, 4mM β-mercaptoethanol, 0.05% of Tween 20, and 20 mM Tris-HCl pH 8.0). Then, the eluates were first incubated with TEV protease at 30°C for 1 h at pH 8.0 and concentrated with lysis buffer after precipitation with ammonium sulfate. The reaction mixture was again subjected to Ni-affinity chromatography. An eluted second flow-through was dialyzed against dialysis buffer (500 mM NaCl, 50% of glycerol, and 20 mM Tris-HCl pH 7.5) in SnakeSkin Dialysis Tubing (Thermo Scientific, USA) at 4°C overnight. Finally, the TAT-Cre protein was sterilized with a Costar Spin-X 8160 Centrifuge tube filter (Thermo Scientific).

### Coomassie Blue Staining and Western Blot Analyses

After centrifugation at 15,000 g for 10 min, the unpurified supernatant and pellet (containing inclusion bodies) were obtained from the cells transformed with the H6-TAT-Cre or H6-E12-TAT-Cre plasmid. The pellets were washed with 20 mM Tris-HCl (pH 7.5), denatured completely with a 1% SDS solution by heating at 85°C, and sonicated until complete solubilization. The solution of a pellet in SDS was combined with the supernatant and designated as “total cellular protein.” The protein levels were measured using the BCA Protein Assay Reagent (Thermo Scientific). Thirty micrograms of proteins were separated by SDS-PAGE in a 12% gel and stained with Coomassie blue or transferred to a polyvinylidene difluoride (PVDF) membrane. The membrane was blocked with 3% bovine serum albumin (BSA) for 1 h and probed with His Probe-HRP (1:5000 dilution in 3% bovine serum albumin, Thermo Scientific) for 1 h. A mouse anti-GAPDH antibody (Thermo Scientific) (1:5000 in Tris-buffered saline containing 0.1% of Tween 20 [TBST]) was incubated with the membrane for 1 h, followed by incubation with a horseradish peroxidase–conjugated mouse antibody (1:5000 in TBST, Thermo Scientific) for 1 h. The blots were developed by means of the Super Signal West Pico Chemiluminescent Substrate (Pierce).

### An In Vitro Assay for Cre Activity Testing

The pMSCV-loxP-DsRed-loxP-eGFP-Puro-WPRE plasmid acquired from Addgene (USA) was linearized by HindIII digestion and reacted with TAT-Cre of different concentrations at 37°C for 1h in 50 μl of a reaction solution containing reaction buffer (33 mM NaCl, 50 mM Tris-HCl, and 10 mM MgCl_2_ pH 7.5) and 300 ng of the linearized plasmid. The solution was heated at 70°C for 10 min to inactivate the Cre, and the DNA fragments were isolated from proteins by phenol extraction and EtOH precipitation. The DNA was analyzed by agarose gel electrophoresis.

### A Cell Viability Assay

293FT cells were cultured in high-glucose DMEM supplemented with 10% of FBS, 1% of a penicillin/streptomycin solution, and 1%of a solution of non-essential amino acids at 37°C and 5% CO_2_. Cell viability was analyzed by the Cell Counting Kit-8 (CCK-8) assay. 293FT cells were seeded at 10⁴/well in 96-well plates coated with a 0.1% gelatin solution and were treated with TAT-Cre at various concentrations for 24 or 48 h. Next, 10% of the CCK-8 colorimetric solution (relative to total reaction volume) was added into each well for additional 4 h cultivation in a humidified incubator. After that, optical density at 450 nm (OD_450_) was measured and normalized to OD_650_.

### Fluorescence Imaging 

293FT cells at 10^5^/well were seeded in 12-well plates coated with a 0.1% gelatin solution and were incubated overnight to attain 60–70% confluence. The cells were transfected with 300 ng of the DsRed-floxed plasmid by means of Lipofectamine 3000 (Invitrogen). After 24 h, we treated pre-sterilized TAT-Cre with the serum-free medium at different doses (0, 1, 2, 4, or 6 μM) for different periods (0, 4, 6, 8, 12, 24, or 48 h). Cell images were obtained at ~480 or 580 nm wavelength using a fluorescence microscope (Olympus, CKX53).

## Results

### Addition of the Poly-Glutamate Sequence Improves Expression of the Soluble TAT-Cre Protein in *E. coli*

**Fig. 1:**
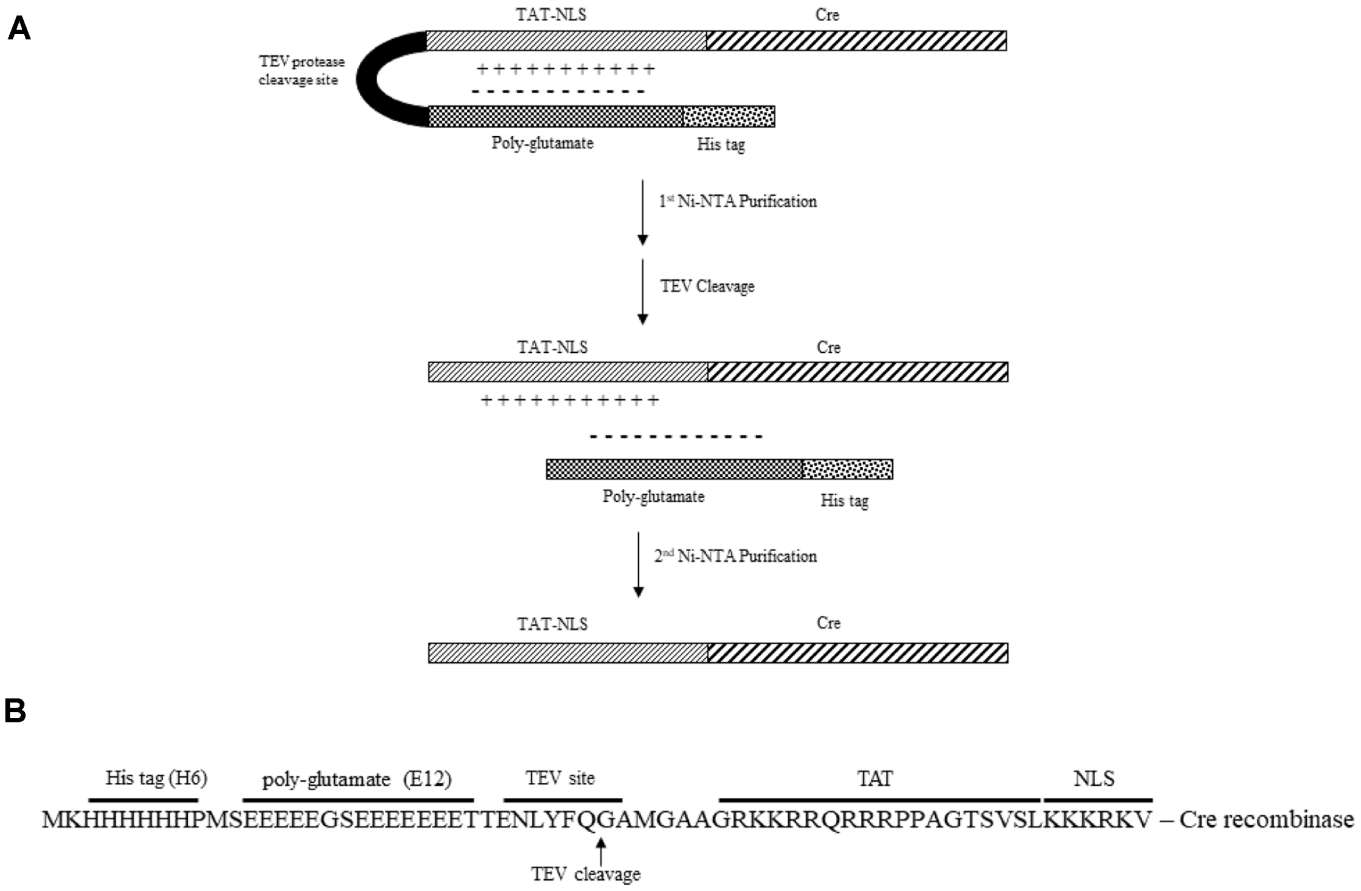
Construction and the amino acid sequence of recombinant H6-E12-TAT-Cre. (**A**) A schematic diagram of construction of the recombinant H6-E12-TAT-Cre. (**B**) The amino acid sequence of poly-glutamate (E12) and TATNLS- Cre cloned into the pETM11 vector, which carries a His tag (H6) and TEV protease cleavage site.

To examine the solubility of the TAT-Cre protein, prokaryotic expression vectors, pETM11-TAT-NLS-Cre as a control and pETM11-E12-TAT-NLS-Cre, were constructed in accordance with the scheme in Fig. 1A. Glycine (G) and serine (S) were added within the E12 domain to prevent possible steric hindrance caused by successive glutamate residues (Fig. 1B). Several amino acid residues, such as glycine, methionine (M), and alanine (**A**), were inserted in the N-terminal TAT tag to ensure flexibility and prevent another possible steric hindrance caused by TEV cleavage (Fig. 1B).

**Fig. 2:**
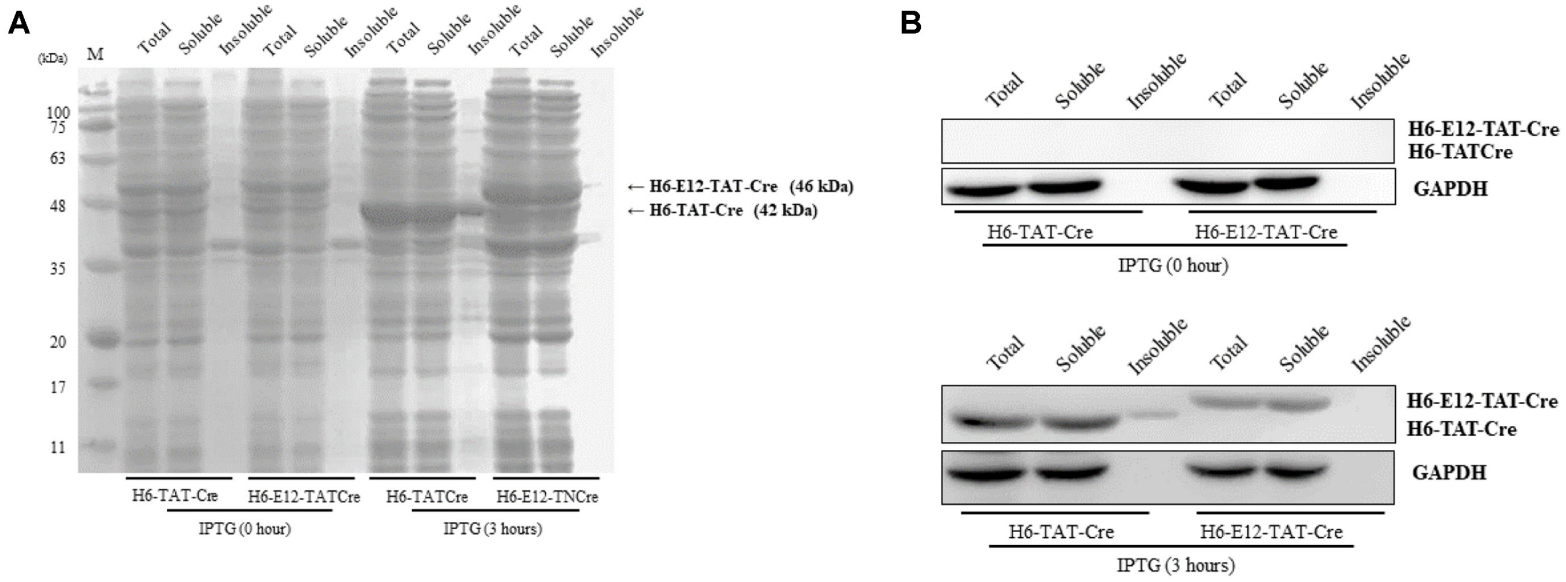
Cellular protein expression of H6-TAT-Cre or H6-E12-TAT-Cre after IPTG induction. (**A**) His-tagged TAT-Cre with or without E12 in total cellular protein is shown on an SDS-polyacrylamide gel stained with Coomassie blue. This staining (on a 12% gel after SDS-PAGE) visualized total cellular proteins of soluble and insoluble fractions from *E. coli* transformed with E12-less H6-TAT-Cre (44 kDa) or H6-E12-TAT-Cre (46 kDa). (**B**) His-tagged TAT-Cre with or without E12 was analyzed by western blotting. The Histagged proteins were detected by an anti–His tag antibody at the 44 kDa (H6-TAT-Cre) or 46 kDa level (H6-E12-TAT-Cre). GAPDH (35 kDa) served as a loading control.

**Fig. 3:**
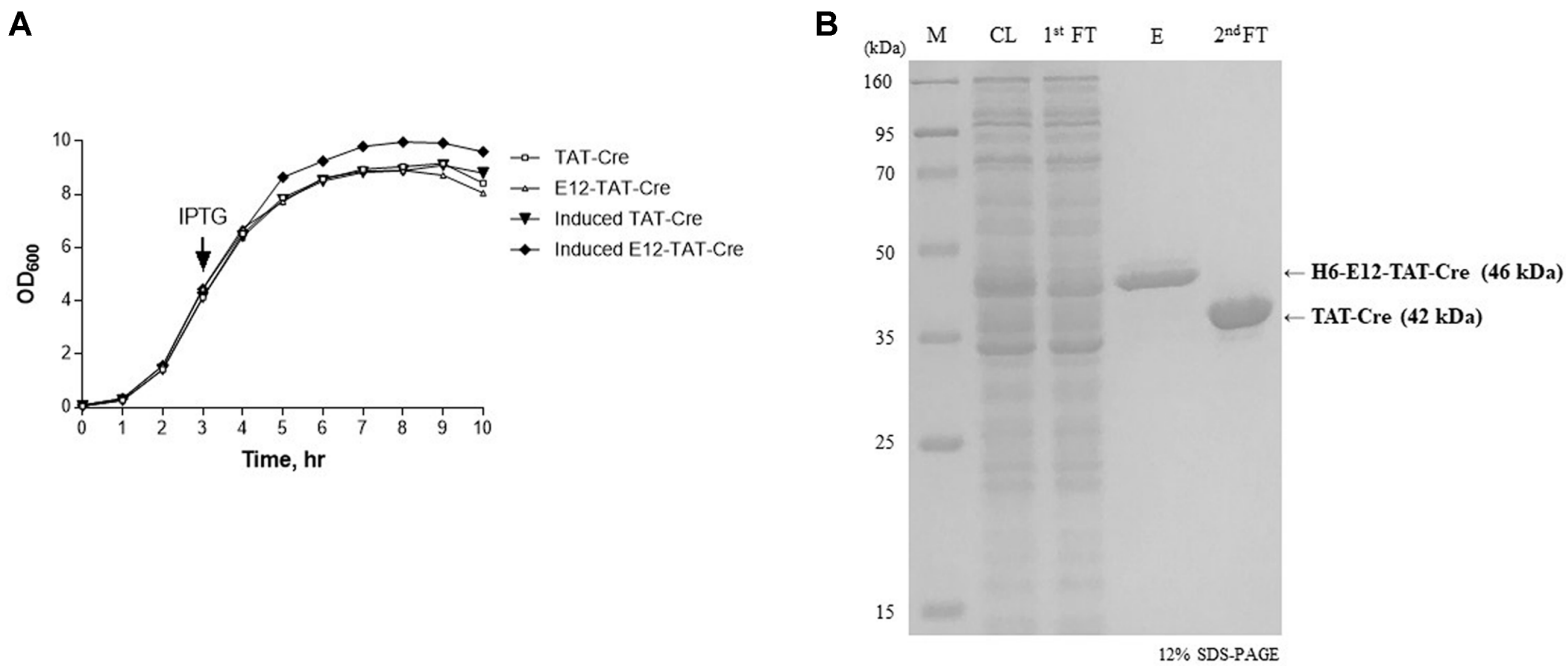
The growth of *E. coli* strains expressing a recombinant protein, and purification of the recombinant TAT-Cre protein. (**A**) The growth of cells transformed with TAT-Cre or E12-TAT-Cre. The growth was evaluated every hour by measuring absorbance at 600 nm until the stationary phase. IPTG induction to express TAT-Cre was started after 3 h incubation at 37°C in the exponential phase. (**B**) Purification of TAT-Cre by Ni-affinity chromatography. Coomassie blue staining was performed on the lysate of cells subjected to IPTG induction. The cell lysate (CL) was obtained from H6-E12-TAT-Cre plasmid–transformed *E. coli* by sonication. The first flow-through (1^st^ FT) did not contain H6-E12-TATCre because His-tagged proteins bind to the Ni-column while other components in the cell lysate pass through the column. The column was washed with a buffer containing 30 mM imidazole, and H6-E12-TAT-Cre (46 kDa) was eluted (**E**) with elution buffer. The eluates were passed through the column again after treatment with TEV protease, resulting in His-tagged H6-E12 and TAT-Cre (42 kDa). The second flow-through (2^nd^ FT) contained TAT-Cre. M indicates molecular size markers.

In case of E12-TAT-Cre protein–expressing cells, there was a significant increase in the portion of the soluble protein (from 82.8 ± 1.6 to 92.8 ± 0.3 mg/ml) and a reduction in the insoluble-protein amount (from 19.4 ± 1.6 to 7.2 ± 0.5 mg/ml) after IPTG induction in comparison with TAT-Cre protein–expressing *E. coli*. The solubility was decreased (from 86.8 ± 0.2 to 83.7 ± 1.5 mg/ml) and total protein levels were decreased drastically in TAT-Cre protein–expressing *E. coli*. In addition, the results of purification revealed that the amount of soluble TAT-Cre (~7.9 mg/l) was increased by addition of the E12 domain when compared to the amount of no E12 TAT-Cre (~4.9 mg/l; Table 1). The corresponding SDS-PAGE and western blot data confirmed that the solubility of TAT-Cre was increased by E12 (Figs. 2A and 2B). These results suggested that the negatively charged E12 sequence of the protein successfully neutralized the TAT sequence rich in positively charged amino acids and subsequently improved the protein solubility. Moreover, it appears that the neutralization led to a higher expression level as well as enhanced solubility of the E12-TAT-Cre protein under the same IPTG induction conditions (Table 1). The cells transformed with the E12-less TAT-Cre plasmid were able to grow up to OD_600_ ≈ 8.8. In contrast, the cells transformed with E12-TAT-Cre plasmid could reach OD_600_ ≈ 9.6. These results suggested that lower expression levels of E12-less TAT-Cre may in part result from the inhibition of cell growth by the positively charged TAT peptide (Fig. 3A).

Purification of TAT-Cre and Measurement of Its Activity The His-tagged E12-TAT-Cre protein (46 kDa) was purified via two consecutive Ni-affinity chromatography steps, and a cleavage reaction by TEV protease was carried out between the two steps (Fig. 1A). Once the cell lysate was prepared, it was loaded onto the Ni-NTA affinity column, and His-tagged proteins bound to the resin in the column. The His-tagged protein was then eluted by higher imidazole concentration. The eluate was next treated with TEV protease, and its recognition/cleavage sequence located between E12 and TAT was cleaved. The resulting digestion products were His-tag/E12 and TAT-Cre recombinase. The resultant product was loaded onto an additional Ni-NTA column. The TAT-Cre recombinase did not bind to the Ni-NTA resin owing to the lack of the His tag, and therefore highly pure TAT-Cre recombinase (42 kDa) was retrieved by collecting flow-through fractions during the second Ni-NTA chromatography while His-tag/E12 was retained within the column (Fig. 3B). Through this purification scheme, highly pure TAT-Cre was obtained, and its yield was 7.9 mg/l of culture, which is significantly higher than that of the control (4.9 mg/l; Table 1).

### Measurement of Activity of Recombinant TAT-Cre in Cell-Free System and 293 FT Cells 

**Fig. 4:**
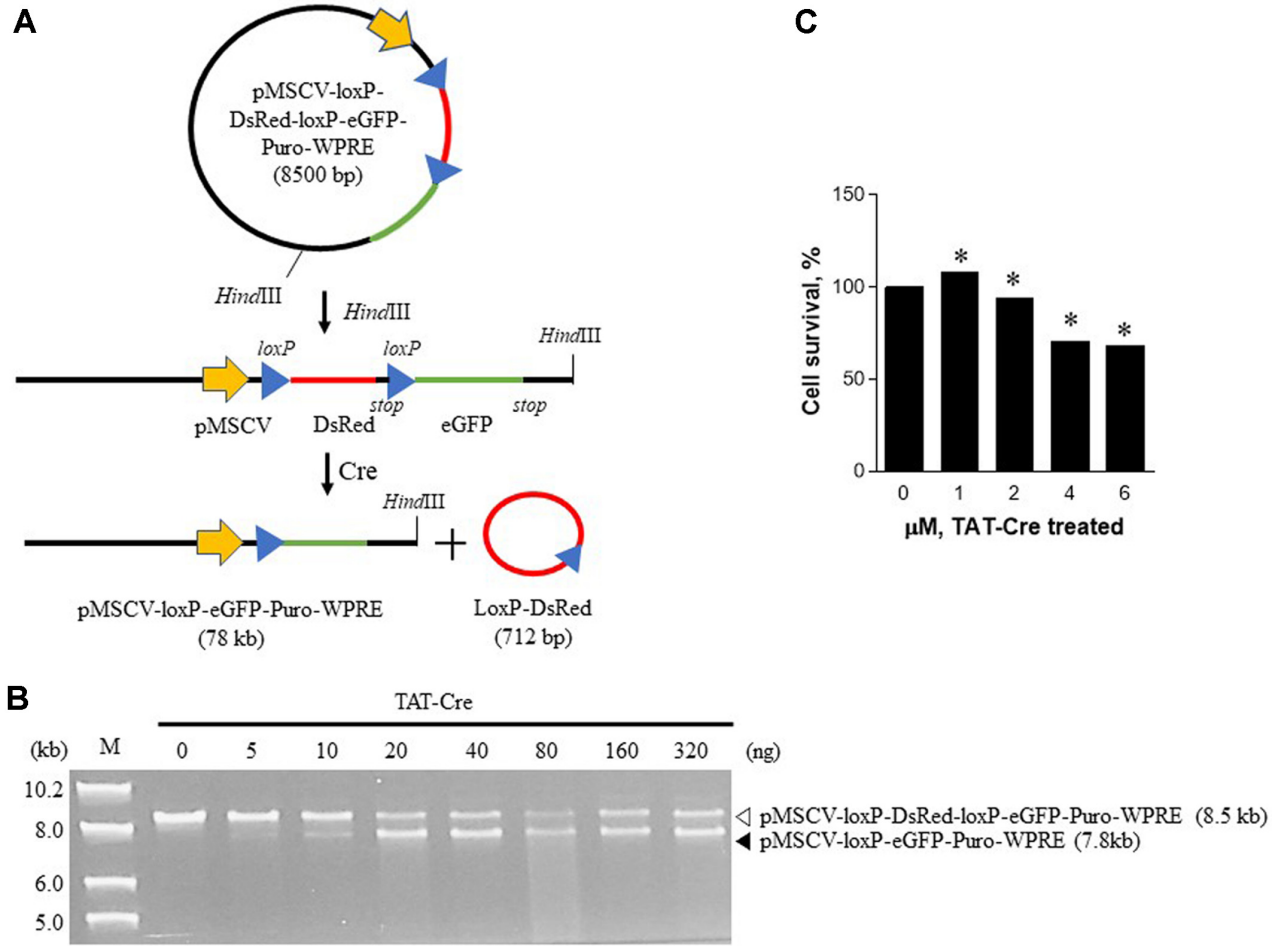
A cell-free assay of Cre recombination activity of recombinant TAT-Cre and the cytotoxicity of TAT-Cre toward 293FT cells. (**A**) The scheme of the TAT-Cre activity assay in cell-free system. pMSCV-loxP-DsRed-loxP-eGFP-Puro-WPRE was linearized by digestion with HindIII and used to assess the recombinase activity of the purified TAT-Cre. (**B**) The cell-free assay of recombinant TAT-Cre activity. A HindIIIlinearized plasmid was reacted with TAT-Cre at various concentrations. The mixtures were incubated at 37°C for 1 h and heated at 70°C for 10 min to inactivate TAT-Cre. Recombination of plasmid DNA was analyzed by agarose gel electrophoresis. M indicates a 1 kb ladder of markers. (**C**) Viability of mammalian 293FT cells exposed to TAT-Cre. The cells were treated with TAT-Cre at various concentrations for 24 h. Cell viability was measured by the CCK-8 assay after the incubation. The results were expressed as a percentage relative to the vehicle treatment control. The data are expressed as mean ± SD; *p < 0.05 as compared with 50% glycerol treatment control (*n* = 3).

To confirm the activity of the purified TAT-Cre, an in vitro recombination assay was performed in a cell-free system. Plasmid pMSCV-loxP-DsRed-loxP-eGFP-Puro-WPRE (8.5 kb) was linearized with HindIII (Fig. 4A). The linearized vector was then used as a substrate of the purified TAT-Cre recombinase. The result indicated that the floxed-DsRed gene was recombined by TAT-Cre recombinase in a dose-dependent manner. Agarose gel electrophoresis yielded a linearized pMSCV-loxP-eGFP-Puro-WPRE band (7,788 bp; Fig. 4B) as a recombination product, whereas another reaction product, tiny circularized floxed-DsRed (712 bp), was not visible in the gel owing to the small size. The LoxP-Cre recombination reaction seemed to be maximized when 40 ng of TAT-Cre was reacted with 300 ng of the plasmid.

**Fig. 5:**
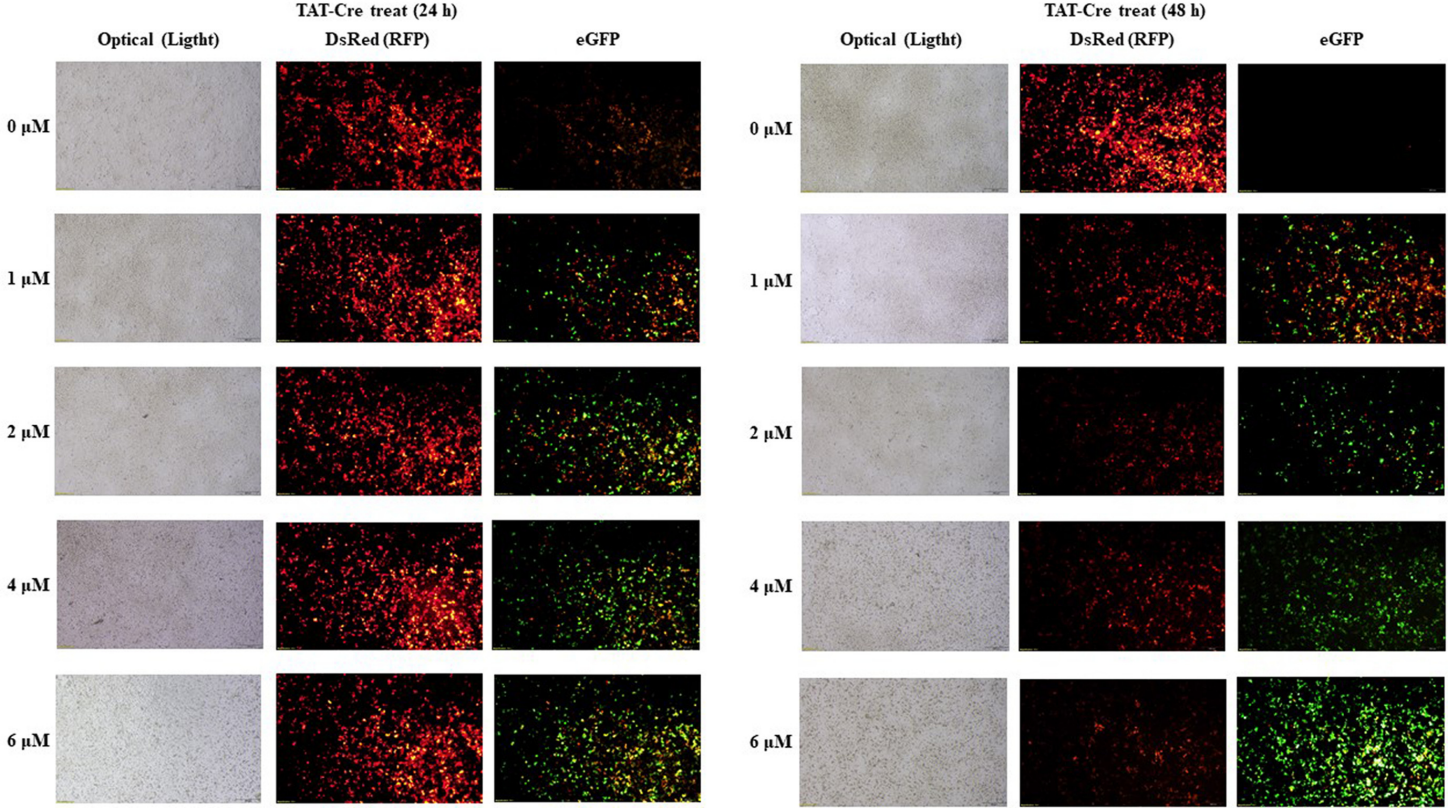
TAT-Cre transduction efficiency in 293FT cells. 293FT cells were transfected with pMSCV-loxP-DsRed-loxP-eGFP-Puro-WPRE via Lipofectamine 3000 for 24 h. The TAT-Cre protein was then administered at various concentrations, and the cells were incubated at 37°C and 5% CO_2_ for 24 or 48 h. The images were taken under a fluorescence microscope.

Next, we examined whether the recombinant TAT-Cre is functionally active in mammalian cells. Because DsRed is flanked with loxP in the pMSCV-loxP-DsRed-loxP-eGFP-Puro-WPRE plasmid, active TAT-Cre can remove DsRed and generate pMSCV-loxP-eGFP-Puro-WPRE via site-specific recombination. 293FT cells were transfected with pMSCV-loxP-DsRed-loxP-eGFP-Puro-WPRE, and the transfected cells started expressing only red fluorescent protein (RFP) because of the proximal promoter when the recombinase treatment was not performed. Without the recombination event, the transcription was started upstream of DsRed, and the translation from the transcript stopped before enhanced green fluorescent protein (eGFP) because there is a stop codon immediately after the DsRFP open reading frame. In contrast, when the recombinase treatment was applied, our enzyme recognized two loxP sites in the vector and removed the DsRed sequence, thereby enabling the expression of eGFP. We tested the TAT-Cre-mediated recombination activity in 293FT cells and cytotoxicity of TAT-Cre, and the assay was performed at various doses and periods of TAT-Cre treatment (Figs. 4C and 5). Noticeable cytotoxicity of TAT-Cre at 4 or 6 μM was observed after 24 h incubation (Fig. 4C). When we treated the transfected cells with Cre-TAT, the green fluorescence signal resulting from eGFP expression (an outcome of TAT-Cre activity) emerged 12 h after TAT-Cre addition, whereas the red fluorescence signal from the DsRed expression persisted throughout the 12 h period shortly after transfection (data not shown). When TAT-Cre was incubated for 48 h, the green fluorescence increased, and the signal was proportional to the concentration of TAT-Cre during the treatment. On the other hand, the red fluorescence diminished after the 48 h treatment, and the signal was inversely proportional to the concentration of TAT-Cre during the treatment, indicating that the in vivo recombination event was successful and was time- and dose-dependent (Fig. 5). These results suggested that the addition of the poly-glutamate tag to TAT-Cre for expression in *E. coli* and purification did not impede either the activities of the TAT and NLS signals or that of Cre recombination.

## Discussion

Protein delivery rather than delivery of a genetic material is considered a safer and useful delivery approach owing to lower toxicity and more efficient transduction. A CPP allows for protein delivery into cells with high efficiency, and CPP-fused proteins have been employed for therapeutic purposes or protein delivery and in some cases have been commercialized [20, 21]. Among CPPs, the TAT peptide has been utilized for protein delivery owing to its ability to penetrate the plasma membrane of all types of mammalian cells except Caco-2 and MDCK cells [22]. Together with an NLS, a cargo protein can be delivered to the nucleus via nuclear pores [23]. A significant amount of a TAT-fused protein expressed in *E. coli* or other bacterial hosts is often destined to inclusion bodies [19]. Researchers have been trying to decrease the amounts of recombinant proteins accumulated in inclusion bodies by various methods including optimization of the incubation temperature and induction conditions [24-26]. In the present study, to overcome the accumulation of a recombinant TAT-Cre protein in inclusion bodies, we attached a negatively charged peptide tag near the positively charged TAT sequence to improve the solubility of the cargo protein in an *E. coli* expression system. We demonstrated that neutralization of the positively charged TAT and of the highly cationic NLS by poly-glutamate (E12) diminished the accumulation of the recombinant protein in inclusion bodies and increased solubility.

We found that TAT-Cre expression increased the amounts of total protein and soluble protein and reduced the quantity of insoluble protein (Table 1). The reason for the greater amount of total protein could be the increased growth biomass of E12-TAT-Cre–expressing *E. coli* (Fig. 3A). This phenomenon is possibly due to a reduction in toxicity that could be related to the highly cationic nature of TAT and NLS amino acid sequences. Indeed, addition of TAT to Cre decreased the solubility and the specific activity of purified fusion protein even with increased yield [19]. In our study, the addition of NLS which has basic properties could contribute to reduction of soluble proteins in cell extracts. In contrast to another report suggesting that expression of a regular TAT-Cre protein increases accumulation of Cre in inclusion bodies [27], we noted substantial expression of soluble recombinant TAT-Cre in *E. coli*. Addition of the E12 domain further improved the solubility and yield of the recombinant TAT-Cre. Whether this strategy is applicable to other proteins should be examined later.

Although the TAT peptide is less toxic than amphipathic CPPs, our findings indicate that TAT-Cre is cytotoxic at higher concentrations (Fig. 4C). However, this problem is due to the toxicity of Cre, not TAT. Prolonged Cre expression has cardiotoxicity, and Cre alone can be cytotoxic when its expression is high enough to affect cell physiology [28]. Because attachment of the TAT peptide to a protein of interest allows for efficient delivery into cells, this strategy has been applied to alleviation of neurotoxicity in a mouse model of Parkinson’s disease [29], stimulation of pancreatic β-cell differentiation [30], and immunotherapy [31]. Therefore, arginine-rich CPPs including TAT have a broader range of applications for research and therapeutic purposes.

Once the E12 sequence together with the His tag is removed by TEV protease during the purification process, the resulting TAT sequence is fully exposed and functional as evidenced by our cell-based assays. We noticed that TAT-Cre recombinase activity in a cell-free system did not perform complete recombination (Fig. 4B). This phenomenon can be due to reversible Cre recombination as evidenced by other reports [27, 32]. In contrast, the recombination was carried out by the enzyme completely in a dose- and time-dependent manner when we assessed the activity in mammalian cells carrying a floxed allele (Fig. 5). When lethality is caused by a tissue-specific gene knockout after Cre-loxP recombination in genetically modified floxed mice, TAT-Cre can be employed for subsequent investigation of the mechanism. To prevent the above lethality, a tamoxifen-inducible Cre system has been devised [33, 34]. Another purpose of the inducible system is prevention of the chronic effects of gene deletion during the embryonic period. Nonetheless, administration of tamoxifen causes adverse effects in addition to induction of Cre. Application of TAT-Cre can prevent this toxicity, and manipulation of primary cells isolated from floxed mice can help to elucidate the underlying mechanism in depth.

In this study, we report that the addition of a polyglutamate domain to TAT-Cre and expression in *E. coli* improves the solubility and yield of the produced recombinant protein. Moreover, we found that the growth rate of *E. coli* expressing E12-TAT-Cre is increased by neutralization of the positive charges of the TAT and NLS sequences. We confirmed that the resulting TAT-Cre is functional in cells and cell-free systems. These results suggest that addition of a polyglutamate domain is a novel way to improve expression and purification of a TAT-fused protein of interest.

## Figures and Tables

**Table 1:** The quantitative analysis of proteins in total cell lysates and soluble and insoluble fractions and solubility of the purified TAT-Cre.

IPTG induction	Sample	Total protein (mg/ml)	Soluble protein (mg/ml)	Insoluble protein (mg/ml)	Purified TAT-Cre (mg/l)	Solubility (%)
0 h	TAT-Cre	105.2±0.9	85.3±2.3	13.0±0.7	-	86.8±0.2
	E12-TAT-Cre	116.3±3.0^*^	93.6±3.1^*^	19.4±1.6^*^	-	82.8±1.6
3 h	TAT-Cre	70.9±7.0^#^	53.9±6.1^#^	10.5±1.7	4.9	83.7±1.5
	E12-TAT-Cre	107.0±5.2^*^	92.5±9.4^*^	7.2±0.5^*#^	7.9^*^	92.8±0.3^*#^

The data are expressed as the mean ± SD of three independent experiments.

^*^p < 0.05 vs. TAT-Cre in same condition, ^#^*p* < 0.05 vs. 0 h IPTG induction.
